# Qualitative study on the shifting sociocultural meanings of the facemask in Hong Kong since the severe acute respiratory syndrome (SARS) outbreak: implications for infection control in the post-SARS era

**DOI:** 10.1186/s12939-016-0358-0

**Published:** 2016-05-04

**Authors:** Judy Yuen-man Siu

**Affiliations:** Department of Applied Social Sciences, Faculty of Health and Social Sciences, The Hong Kong Polytechnic University, Hung Hom, Hong Kong

**Keywords:** Health behavior, Community and Public Health, Behavioral change, Facemask, Sociocultural meaning, Hong Kong

## Abstract

**Background:**

The clinical importance and efficacy of facemasks in infection prevention have been documented in the international literature. Past studies have shown that the perceived susceptibility, the perceived severity of being afflicted with life-threatening diseases, and the perceived benefits of using a facemask are predictors of a person’s use of a facemask. However, I argue that people wear a facemask not merely for infection prevention, and various sociocultural reasons have been motivating people to wear (and not wear) a facemask. Facemasks thus have sociocultural implications for people. Research on the sociocultural meanings of facemasks is scant, and even less is known on how the shifting sociocultural meanings of facemasks are related to the changing social environment, which, I argue, serve as remarkable underlying factors for people using (and not using) facemasks. As new infectious diseases such as avian influenza and Middle East Respiratory Syndrome have been emerging, threatening people’s health worldwide, and because facemasks have been documented to have substantial efficacy in the prevention of infection transmission, understanding the sociocultural meanings of facemasks has significant implications for public health policymakers and health care providers in designing a socially and culturally responsive public health and infection control policy for the community.

**Methods:**

A qualitative research design involving the use of 40 individual, in-depth semistructured interviews and a phenomenological analysis approach were adopted.

**Results:**

The sociocultural meanings of the facemask have been undergoing constant change, from positive to negative, which resulted in the participants displaying hesitation in using a facemask in the post-SARS era. Because it represents a violation of societal ideologies and traditional Chinese cultural beliefs, the meanings of the facemask that had developed during the SARS outbreak failed to be sustained in the post-SARS era.

**Conclusion:**

The changes in meaning not only influenced the participants’ perceptions of the facemask but also influenced their perceptions of people who use facemasks, which ultimately influenced their health behavior, preventing them from using facemasks in the post-SARS era. These findings have critical implications for designing a culturally responsive infection prevention and facemask usage policy in the future.

## Background

The outbreak of Severe Acute Respiratory Syndrome (SARS) in 2003 marked a critical turning point for public health development in Hong Kong. The SARS outbreak caused 1755 infection cases, with 299 deaths in Hong Kong, yielding a fatality ratio of 17 % [1]. The outbreak has facilitated the development of public health at the institutional level, but it has also enabled the Hong Kong population to become aware of the importance of personal hygiene and infection prevention at the community level. Facemask use, which was a remarkable social and health behavior in Hong Kong during the SARS outbreak, was widely recognized by the Hong Kong population for the first time. Since then, the Hong Kong population has become familiar with the use of facemasks in public spaces.

### Significance

The clinical importance and efficacy of facemask infection prevention are documented in the international literature [2, 3]. Past studies have shown that the perceived susceptibility, the perceived severity of being afflicted with life-threatening diseases, and the perceived benefits of using a facemask are predictors of people’s facemask use [2]. However, I argue that people use a facemask not only for infection prevention. Various sociocultural reasons motivate people to use (and not use) a facemask, which has sociocultural implications for people. However, research on the sociocultural meanings of facemasks is scant, and even less is known on how the shifting sociocultural meanings of facemasks are related to the changing social environment, which, I argue, serve as critical underlying factors for people’s use (and the absence of use) of a facemask. Because of the effects of globalization and well-developed international transportation, epidemic outbreaks are no longer confined to a single country. As new infectious diseases such as avian influenza and Middle East Respiratory Syndrome (MERS) have continued emerging, threatening people’s health worldwide, and because facemasks have been documented to have significant efficacy in the prevention of infection transmission, therefore, understanding the sociocultural meanings of the facemask has critical implications for public health policymakers and health care providers in designing a socially and culturally responsive public health and infection control policy for the community.

Symbolic representation, or symbolism, is a popular area of study in the humanities. Medical instruments such as a stethoscope and white coat often convey symbolic implications [4, 5], affecting people’s identity, emotions, and behavior [5, 6]. These symbolic implications influence how people perceive others who are associated with these objects, which influences how they react to the objects. In a similar manner, the facemask also has symbolic implications for people. These implications influence how people perceive facemasks, how people perceive those who use them, and how people respond to facemask use. These factors in turn affect people’s adoption of preventive health behavior regarding facemask use. Moreover, these symbolic implications undergo constant change because of the different and changing sociocultural environment. Facemasks carry different symbolic implications for people in different societies, and hence, different perceptions and interpretations exist regarding facemask use, resulting in different health behaviors among communities. For example, concerning the 2015 MERS outbreak in South Korea, the difference in attitude between South Koreans and the Hong Kong population toward facemask use not only generated cultural clashes between the two populations [7–10], but could also lead to different outcomes in epidemic control. However, literature on the symbolic implications of facemasks for people is scant; how the mechanism of such changes in symbolic meaning is triggered remains unknown. Because of this research gap, this study investigated the sociocultural meanings of facemasks in Hong Kong during the SARS outbreak and in the post-SARS era, showing how the changes in the symbolic implications of the facemask have occurred. This study also examined how these changing symbolic implications can influence people’s adoption (and repudiation) of facemasks when used as a preventive health measure.

## Methods

For this study, a qualitative research design involving individual, in-depth semistructured interviews was adopted. A total of 40 participants were recruited from and interviewed at one private-practice primary health care clinic during the peak season of influenza A (H3N2) in January 2015 in Hong Kong through purposive sampling. A phenomenological approach was adopted for data analysis.

### Ethics, consent, and permission

The Committee on the Use of Human and Animal Subjects in Teaching and Research at Hong Kong Baptist University approved this study. Permission for participant recruitment and interview implementation was sought prior to the study from the owner of the primary health care clinic, who is a private-practice general practitioner. Participation in the study was entirely voluntary. I provided all the participants with an information sheet written in their native language—traditional Chinese—informing them of the purpose, nature, and procedure of the study. Verbal explanations and clarifications were also provided. I obtained a signed consent form from each participant, and all participants were aware that they were free to withdraw from the study without having it affect their current and future treatment received at the clinic.

I conducted all interviews in an examination and procedure room in the clinic to protect the participants’ privacy. The interviews were audio-recorded with the participants’ consent. To further protect the participants’ privacy and confidentiality, no participant identifiers were mentioned during the interviews. I maintained all data and field notes in locked cabinets and treated them with strict confidentiality. To further minimize the risk of potential identification, I represented each participant with a code in the data and interview transcripts. The digital audio recordings of the interviews were destroyed once the interviews were transcribed.

### Consent to publish

The participants were informed thoroughly on the use of their provided data in academic publications, with all the personal identifications removed to ensure privacy.

### Reflexivity of facemask use

My knowledge and understanding of the use of facemasks can be traced to the SARS outbreak in 2003, when I was a Masters student conducting research in the Department of Anthropology at The Chinese University of Hong Kong. In the beginning of the SARS outbreak, most people in Hong Kong, myself included, were unaware of the importance of using a facemask. Facemask use was extremely rare for people in Hong Kong and could be perceived as a display of strange behavior, and the person using it could be viewed as a highly infectious patient warranting social seclusion. Although a few people had been observed using a facemask in public spaces at the beginning of the outbreak, they were often secluded. In one case, I encountered a woman who was using a facemask, and my immediate response was to walk away from her.

The facemasks that were being used in the beginning of the SARS outbreak did not meet clinical standards. Rather than a surgical facemask, many other types of facemasks (e.g., paper and active-carbon masks) emerged in the market.

In a manner similar to that of most Hong Kong citizens, I first became aware of the importance of using a facemask in public spaces when medical scientists announced the severity of the nosocomial outbreak of SARS at Prince of Wales Hospital, which was an epicenter of the outbreak, at a press conference. All of the professors at the Faculty of Medicine in The Chinese University of Hong Kong used facemasks at the press conference, which visually reinforced the severity of SARS. Alarmed by the severity of the outbreak, my family began to scramble for facemasks, as did the rest of Hong Kong. According to the official recommendations of medical professionals, my family aimed to obtain three-layered surgical facemasks because N95 respirators had been depleted during the outbreak.

Similar to other Hong Kong residents, I engaged in facemask scrambling with my family, and used a facemask at all times in public spaces throughout the SARS outbreak, from March to July of 2003. Moreover, at the community level, different social institutions also exerted a strong mode of control over my facemask use during those months. All students were required to use a facemask in schools and in universities during the outbreak. Failure in using a facemask in the university that I was studying would mean that these noncomplying students were to be prohibited from attending lectures. Using a facemask during the SARS outbreak was widely perceived as an infection-preventive measure; furthermore, using a facemask in public areas was perceived as a new social norm, and a person would be subject to discrimination if he or she failed to comply. In one case, I encountered a neighbor in the lift who just used tissue to cover up her nose and mouth, and I immediately walked out from the lift and stared at her at the same time. My values and beliefs toward facemasks have changed since the SARS outbreak. Before the outbreak, I did not have any knowledge of using facemasks. My impression of those who used a facemask was negative before the outbreak, and I perceived that these people were either strange or mentally ill. The SARS outbreak, however, has changed my perceptions of facemask use and instilled in me more positive perceptions of this behavior.

As a public health researcher, I am aware that the primary purpose of facemasks is infection containment and that they should be used by those who are suspected of or have been diagnosed with an infectious disease. I have been perceiving facemask use as a civic responsibility when one falls ill. However, as an anthropologist, I am also aware that the perceptions of facemask use are never static for one community, but rather, these meanings undergo constant change over time. Because of different life encounters and experiences, people would have varying perceptions of facemasks, and these perceptions can influence whether they use a facemask, akin to what I have been experiencing in my life also influencing my perception of facemasks, and in turn influencing my facemask use behavior. Instead of representing sick people without a facemask as not exercising their civic responsibilities, this study examined the shifting cultural meanings of facemask use in Hong Kong from the SARS outbreak in 2003 to the influenza A (H3N2) outbreak in January 2015 and how these shifting cultural meanings could have implications for infection control in the future.

### Data collection

The 40 participants were selected through purposive sampling, conducted in accordance with the following inclusion criteria, from a private-practice primary health care clinic in Hong Kong. Although past studies have documented that 17 interviews suffice for achieving data saturation [11], this study comprised 40 interviews to yield greater confidence in the data. In addition, data saturation was achieved using no new data and information that could be collected from the interviews [12]. To investigate the changing meaning of facemasks in Hong Kong from the SARS outbreak to the study period, the participants were selected based on their experience of facemask use during the SARS outbreak, but not in the post-SARS era as well as at the sampling venue, despite having experienced respiratory symptoms. In-depth semistructured interviews were conducted with individual participants during the peak season of influenza A (H3N2) in January 2015, because sampling during this period ensured the efficient recruitment of suitable participants.

With the consent of the primary health care clinic, I visited the clinic for 14 consecutive days throughout the physician’s consultation hours to identify, recruit, and interview the participants. Those who did not use a facemask were approached, and they were asked the following questions pertaining to the sampling criteria, so that I could identify if they were suitable for the interviews:

1. Are you suffering from any respiratory symptoms today?
2. Did you use a facemask during the SARS outbreak in 2003?
3. Are you currently using a facemask?
4. Have you been residing in Hong Kong since the SARS outbreak in 2003 until today?

To fulfill the sampling criteria and enable an investigation into the shifting sociocultural meanings of facemask use since the SARS outbreak in 2003 until the influenza A (H3N2) outbreak in January 2015, the participants were required to answer “yes” for Questions 1, 2, and 4 and “no” for Question 3.

To minimize potential disturbances of clinical operations and participants, those who fulfilled the sampling criteria and who consented to the interview were interviewed immediately in another examination and procedure room within the clinic after they completed their consultation and received their prescription medication. Conducting the interviews after the participants had completed treatment and received their prescription also assured the participants that the interviews did not affect their treatment. The participants were assured of interview confidentiality and that their viewpoints would not affect their future consultations and treatment in the same clinic.

An interview question guide was developed according to past studies on the use of facemasks as well as my personal long-time observations of the use of facemasks in Hong Kong. Regarding the investigation into the participants’ views on facemasks as well as their use of facemasks during and after the SARS outbreak, the interviews were conducted to examine the changes in the use and meaning of facemasks from the SARS outbreak in 2003 until the post-SARS era in 2015. The interview questions included the following:

1. Why did you use a facemask during the SARS outbreak?
2. For how long have you not used a facemask? Why?
3. Did you use a facemask after the SARS outbreak?
  If yes, what was the occasion? And why?
  If no, why?
4. Why are you not using a facemask now?
5. How did you view facemasks during the SARS outbreak?
6. How do you view facemasks now?
7. How did you view people who used facemasks during the SARS outbreak?
8. How do you view a person using a facemask now?
9. Who needed to use a facemask during the SARS outbreak?
10. Who needs to use a facemask now?
11. Can you share your life story, experience and feelings during the SARS outbreak?
12. How do these experience and feelings affect you in the SARS outbreak?

The interview question guide was used throughout the interview process to guide the discussion and to ensure that the interviews did not deviate from the research questions. I conducted all of the interviews to maintain consistency and to ensure interview quality. This approach also minimized the risk of data variations and data flaws that might have resulted from introducing another interviewer. Follow-up probing in the interviews was performed in response to the participants’ answers to collect additional in-depth data from the participants. All of the interviews were conducted in Cantonese Chinese, which is the native language of the participants as well as my own, to ensure free expression of the participants’ views. The interviews were audio-recorded with the participants’ consent. The duration of each interview was 1 h and 10 min to 1 h and 35 min. Each participant was presented with a supermarket coupon of HK$100 as an acknowledgment of their participation to compensate them for their time.

### Data analysis

To ensure that the interviews yielded the most data, a quick data analysis was performed during the interviews to ascertain the obtained knowledge and to determine areas that required further exploration [13]. A student assistant transcribed the interviews verbatim, and I subsequently translated the interview transcripts into English. Backtranslation was performed by another bilingual student assistant to ensure the translated interviews did not distort the original meaning of the participants.

A phenomenological approach was adopted to analyze the interview data and to discover the patterns and structures of phenomena that are the lived experiences of people [14]. Using a phenomenological approach, this study examined the lived experiences of facemask use by the participants as well as their health perceptions and behaviors in response to infectious diseases since the SARS outbreak in 2003 until the influenza A (H3N2) outbreak in January 2015. A procedure involving intuiting, analyzing, and describing was conducted [14]. By learning the facemask usage experiences of the participants, and by examining the structure of facemask usage by examining the participants’ experiences, the manifestations of the facemask usage phenomenon as a meaning of the experiences with Hong Kong’s sociocultural structures were recognized and affirmed in the analytical process [14]. The meaning of facemask use as experienced by the participants was thus determined.

Major themes in the data were identified, which involved segmenting the interview transcripts into individual meaning units, collapsing them into categories, and determining themes through a process involving abstraction and constant comparisons. A coding table was developed that identified themes, categories, and codes with supporting evidence in the form of interview quotes; this was achieved in line with the inductive coding process to enable the discovery of patterns of behaviors and thoughts [15]. Recurrent codes and themes were noted and highlighted. New thematic codes that emerged from the data were added to the coding table. Memos were used to record ideas and commentary during coding. The analytical procedures, coding, and findings were documented in the codebook to ensure the consistency and accuracy of the analyzed data. Data saturation was achieved.

### Rigor

After the interviews were translated from Chinese to English, backtranslation from English to Chinese was performed to ensure that the translated interview quotations did not distort the original meaning of the participants. To avoid researcher bias, motivation, or interest in the analysis, and to establish credibility and neutrality, direct interview quotations were included in the coding analysis, enabling the findings to be grounded in the data. Because data collection and analysis were conducted by a single researcher, coding and recoding of the transcripts were performed to ensure reliability and confirmability and to ascertain that the coding and categories were free of ambiguity, overlaps, and lack of clarity. Recoding was conducted 1 month after the first coding session as cross-checking to enhance the validity and reliability of the data and findings and to ensure the elimination of subjectivity and bias.

## Results

### Participants

All 40 participants were patients at a private-practice primary care clinic. They were ethnically Hong Kong Chinese people comprising 29 women and 11 men aged between 22 and 70 years at the time of this study. To examine the changing meanings of facemasks in Hong Kong from the SARS outbreak until the study period, the participants were purposively sampled according to the following inclusion criteria: (a) They suffered from respiratory symptoms at the time of sampling, (b) they did not use a facemask at the sampling venue, and (c) they had used a facemask during the SARS outbreak in 2003.

Totally, 17 participants claimed to be healthy and not suffering from chronic conditions, whereas the remaining 23 participants suffered from various chronic conditions, including hypertension, diabetes mellitus, gout, liver disease, and heart disease. These 23 participants were receiving long-term follow-up treatment either in public hospitals or at the private clinic in which sampling was performed. All of the participants visited the clinic because of respiratory symptoms during the sampling period: 15 participants were diagnosed with influenza, 21 with upper respiratory tract infections, 3 with bronchitis, and 1 with pneumonia.

All the participants had experience with using a facemask during the SARS outbreak. However, most of them had not been using a facemask since the outbreak ended. Although a few participants had used a facemask for infection prevention at the onset of the influenza A (H1N1) outbreak in Hong Kong in 2009, none of them had used a facemask because of their respiratory symptoms after the SARS outbreak. The shifting sociocultural meanings of facemask during the SARS outbreak and post-SARS era in Hong Kong, which are to be illustrated in Table 1 and in the themes below, explain the changing pattern in facemask using behavior among the participants.

**Table 1:** Comparison of the sociocultural meanings of facemasks during the SARS outbreak and post-SARS era in Hong Kong

	During the SARS outbreak	In the post-SARS era
Perceived function of facemasks	Primarily used for infection prevention	Primarily used for infection prevention
Sociocultural meaning of facemasks	Perceived as a new social norm	Perceived as a sign of people with negative attributes
	Perceived as a form of civic responsibility	Perceived as a medium for inviting stigmas and teasing
	Perceived as symbolic support for health care providers	Perceived as a sign of weakness
	Perceived as a tool for achieving a sense of control and security	Perceived to hinder recovery
Perceived nature of facemasks	Medical professionals’ advice was critical for the participants’ choice of an appropriate facemask	Toxic to skin

All of the sampled participants were employed in different sectors, including administrative and executive, sales and retail, commerce and finance, civil service, education, social welfare, hotel management, and information technology sectors.

### Meaning of facemasks during the SARS outbreak

All of the participants had used a facemask during the SARS outbreak. In addition to practical concerns regarding infection prevention, sociocultural controlling mechanisms also motivated the participants to use a facemask during the SARS outbreak. Using a facemask during the outbreak was a new social norm and was perceived as a show of support for health care providers and a display of civic responsibility. Using a facemask also enabled the participants to achieve a sense of control and security in the pervasive social atmosphere of uncertainty.

#### Facemask for infection prevention

The predominant reason for using a facemask during the SARS outbreak for all the participants was their concern regarding SARS infection prevention. One participant recalled the following:

SARS was so horrible. It was deadly, but highly transmittable. I could not ask other people to use a facemask before coughing and sneezing. Therefore, I wore a facemask to prevent myself from getting infected. This was the only thing that I could do as a prevention against SARS.

[Female participant (P2), 56 years old]

Using a facemask in public spaces was perceived as a necessary SARS infection prevention measure for all of the participants during the SARS outbreak. One participant added the following:

Wearing a facemask was necessary when you were in public places during [the] SARS [outbreak]. You could never know who the hidden virus carriers were, but they could still spread the virus and infect others when they spoke, coughed, or sneezed. Moreover, you could never know if the places you went to had been contaminated by the SARS virus. If you did not wear a facemask in public, then you would be at a much higher risk of getting infected.

[Female participant (P16), 59 years old]

#### Facemask use as a new social norm

Using a facemask was a new social norm in Hong Kong during the SARS outbreak, and all of the participants perceived this social norm to be a powerful motivator for them in using a facemask. The participants commonly perceived that failing to use a facemask in public during the SARS outbreak could make them potential victims of discrimination. One participant noted the following:

SARS has changed the culture of facemask use in Hong Kong. Before [the] SARS [outbreak], no one would use a facemask. I did not even know where I could buy a facemask before [the] SARS [outbreak]. However, when SARS hit, using a facemask was just like wearing clothes in public, and it was a norm in society. Everyone in Hong Kong wore a facemask during [the] SARS [outbreak]. If you did not wear a facemask, you would be given a dirty look and be discriminated against. No one dared not to wear a facemask at that time.

[Male participant (P5), 52 years old]

Failure to conform to the new social norm of using a facemask in public during the SARS outbreak could have invited others to exercise social seclusion. One participant indicated the following:

In the beginning, I was very reluctant to wear a facemask, because I was worried that this would scare people away. However, no one was willing to talk to me if I did not wear one. My friends and colleagues maintained a great distance from me when I spoke. Others just ran away when they noticed that I was not wearing a facemask on the streets or during public transportation. I then recognized that it was a necessity to wear a facemask when I was outside my home. After I wore a facemask, my friends and colleagues no longer avoided me; others were willing to stand or sit next to me when I used public transportation.

[Male participant (P10), 42 years old]

The mass media played a critical role in normalizing the use of facemasks and in reinforcing the new sociocultural norm of using a facemask in public. Either they achieved these conditions by using words implying moral judgment in their reports, condemning those who did not use a facemask, or blaming and stigmatizing those who were infected with SARS. One participant indicated the following:

News reports often referred to SARS as “super pneumonia” and referred to those who were infected with SARS as “super virus spreaders.” SARS patients were frequently regarded as spreading the deadly virus to others. With these reports, you would soon realize that if you were infected with SARS, then you would be labeled with these negative attributes. Many news reports also kept reporting about doctors’ advice on using facemasks, and these reports also negatively labeled those who did not wear a facemask in public as weird and lacking social consideration.

[Female participant (P21), 45 years old]

Photographs displaying people using facemasks in media reports were also a considerable force in reinforcing the new social norm of using facemasks in public. Those who failed to use facemasks were labeled as “abnormal,” and through such reports, the failure to use facemasks in public was transformed into a form of social deviance. One participant stated the following:

During [the] SARS [outbreak], all the newspapers and television news headlines were about SARS. The photos in these reports often showed people wearing facemasks. These photos were really eyecatching because everyone in these photos was wearing facemasks. In the beginning, I did not think I needed to wear one. However, those who did not wear a facemask were portrayed as aliens or monsters in these news reports. Then I learned that I should wear a facemask.

[Female participant (P17), 48 years old]

#### Facemask use as a display of civic responsibility

With the new social norm of using a facemask in public during the SARS outbreak, all of the participants were motivated to use a facemask to avoid being discriminated against. This new social norm contributed to the development of a novel definition of civic responsibility during the SARS outbreak, which caused nearly all of the participants to perceive that using a facemask in public was a social responsibility. One participant related the following experience:

Wearing a facemask was a way to tell others that you were exercising civic responsibility and that you cared a lot about Hong Kong. You did not want to spread the virus to others, so you wore a facemask. You wanted to tell others that you were on the same team with other Hongkongers in fighting against SARS, so you wore a facemask. Wearing a facemask was a sign of solidarity and civic responsibility at that time. I think these meanings of wearing a facemask were more important, so a facemask was not merely for protecting you against SARS. It meant a lot more than that.

[Female participant (P34), 52 years old]

Those who failed to use a facemask in public during the SARS outbreak were perceived as lacking social responsibility. One participant stated the following:

I think that those who did not wear a facemask were not being socially responsible. You could never know if you got infected [with SARS]; no symptoms would emerge during the 10-day incubation period, but you could infect others if you did not wear a facemask, not to mention that if a facemask was really useful for preventing the spread of SARS, wearing a facemask was just like showing your social responsibility and concern to others. You may be able to recover after getting infected of SARS, but you may kill others if you do not wear a facemask. My wife died of SARS because of these irresponsible people.

[Male participant (P26), 64 years old]

#### Facemask as symbolic support for health care providers

Health care providers played a crucial role in motivating most of the participants to use a facemask during the SARS outbreak. Their advice to use a facemask during the SARS outbreak served as a powerful motivator for the participants to use a facemask. One participant indicated the following:

During [the] SARS [outbreak], information on [the use of] facemasks bombarded us daily. Those “women’s television programs” often interviewed different doctors on the use of facemasks and also compared the advantages and disadvantages of each type of facemask. These doctors also mentioned how to select the most appropriate facemask, how to wear it, and how to use it effectively and hygienically in these programs. I encountered this information every day, and this made wearing a facemask a necessity at the time.

[Female participant (P38), 62 years old]

Because of the image of facemasks and the suggestion of health care providers to use them, the participants began to feel comfortable with and became motivated to use facemasks. Using facemasks was also perceived as a show of support for health care providers during the SARS outbreak. One participant expressed the following:

During [the time of] SARS, all medical professors, doctors, and nurses were wearing a facemask. Facemasks were just like a stethoscope and white coat, and it was a symbol of these health care heroes. Therefore, if they wore facemasks, I also felt confident with the efficacy of wearing one. Besides, I wanted to show my support for doctors and nurses; I thought wearing a facemask was the best way of showing my support for them, because I could then minimize the possibility of getting infected with SARS. If I got infected, I would increase their workload. Therefore, I wore a facemask throughout the outbreak, because I did not want to increase their burden. This should be the best way of supporting them.

[Female participant (P31), 58 years old]

#### Facemask as a tool for achieving a sense of control and security

Facemasks were imbued with a mysterious power in that they provided a sense of security for more than half of the participants during the SARS outbreak. Moreover, facemasks provided a sense of control to the participants in an atmosphere of pervasive uncertainty that was prevalent in Hong Kong. One participant recalled the following:

I felt a strong sense of uncertainty with my life during [the] SARS [outbreak], because I did not know when I would be infected. So many people were getting infected every day, and so many people died from SARS daily. At the time, I had a very strong feeling that I would definitely get infected sooner or later; it was just a matter of time, and death was near to me. I did not know what I could do. The only thing that I could do at the time was to wear a facemask. With a facemask, I felt much more secure. I did not know what a facemask could do to save me from [getting infected with] SARS, but I just felt that wearing it was the only way to save my life.

[Male participant (P27), 59 years old]

The lack of information on this new infectious disease also intensified the sense of uncertainty among the participants during the SARS outbreak. Using a facemask was thus the only action they could adopt to overcome this sense of uncertainty. Another participant indicated the following:

I felt so helpless during [the] SARS [outbreak]. SARS was so mysterious that I really did not know how it could be transmitted. I just knew that SARS was extremely deadly, and it was highly contagious. Doctors and nurses still got infected, even though they used protective gowns and facemasks. People living in Amoy Gardens were all infected, even though they were not living together in the same flat. I could never know who a virus carrier was when I was on the streets. I could never know if the air contained the SARS virus as well. There was so much that was unknown about SARS, and there was so little that we could do and control. However, I still had to do something, and wearing a facemask was the only thing that I could do at the time. It gave me a sense that I could at least have some control over my life.

[Female participant (P11), 51 years old]

### Meaning of facemasks in the post-SARS era

Although using a facemask in public was a social norm in Hong Kong during the SARS outbreak, this social norm has been diminishing gradually in the post-SARS era because of the shifting meaning of the facemask, which prevented people from using it. Using a facemask was perceived as uncomfortable. Moreover, it was viewed as a hindrance to recovery, and a stigma was present against people who used facemasks, who were subject to teasing and discrimination. Negative attributes were associated with people who used them, and the perceived harm to skin as well as the violation of traditional Chinese cultural expectation on certain gender made the participants reluctant to use a facemask in the post-SARS era.

#### Facemask use as discomfort

Sensing physical discomfort was the most powerful reason for the participants choosing not to use a facemask when they suffered from respiratory tract infections in post-SARS Hong Kong. All of the participants indicated that using a facemask when sick often made them feel even more uncomfortable. One participant expressed the following popular concern:

Even for a healthy person, wearing a facemask can make you feel difficulties and discomfort in breathing; so asking a sick person to wear a facemask is quite inhumane to me, since this will make him or her feel even sicker. When I get a cold or flu, my nose is blocked, and I feel even sicker when I wear a facemask, because the facemask makes it even harder for me to breathe. I also need to speak louder when wearing a facemask, but I may have a sore throat when I’m sick. Wearing a facemask is very hot too, especially in the summer.

[Male participant (P4), 22 years old]

#### Facemask for infection prevention

Similar to perceptions that were commonplace during the SARS outbreak, infection prevention was still perceived as the main reason for using a facemask for all of the participants in the post-SARS era. One participant stated the following:

I often believe that those who wear a facemask are actually healthy; they wear a facemask because they are afraid of being infected. Those who are coughing and sneezing often do not wear a facemask. Facemasks are now used by healthy people to stop themselves from getting infected but are not used by sick people to prevent themselves from spreading viruses to others.

[Female participant (P9), 43 years old]

According to the participants’ perception that facemasks were used mainly for infection prevention, many participants perceived that the purpose of facemask use was limited when they were sick. One participant indicated the following:

If I am sick, what is the point of me wearing a facemask? A facemask is used to protect you from getting infected, and wearing a facemask will not make any difference for a sick person to recover faster, so why should I bother to wear a facemask if I am sick?

[Female participant (P35), 32 years old]

#### Facemask as a hindrance for recovery

Nearly all of the participants perceived that using a facemask when sick could hinder their recovery. This belief prevented them from using a facemask when they became infected with a disease. One participant stated the following:

It is not good to use a facemask when you catch a cold or flu, because it covers your nose and mouth. When you cough and sneeze, the bacteria and virus will stick to your facemask, and they will have nowhere to go. You will then breathe in the bacteria and virus again, and you can never recover. You have to let the bacteria and virus out of your body, so it is not good to use a facemask when you are sick.

[Female participant (P24), 68 years old]

The popular Chinese belief of having to infect others to recover was deeply embedded in more than half of participants’ minds. Because using a facemask prevented one from passing the virus to others, the participants perceived that using a facemask could prevent a quick recovery. One participant explained this belief as follows:

It is often said in traditional sayings that if you want to recover quickly from a cold or flu, then you have to infect others. You will get well when others get sick from you. Therefore, if you wear a facemask, it will prevent you from infecting others, so you will find it difficult to recover. I know this is bad, but I think it is normal for everyone to hope for a quick recovery. Therefore, I tend not using a facemask when I am sick.

[Male participant (P23), 65 years old]

#### Facemask as a medium for inviting stigmas and teasing

In addition to practical concerns, facemasks also conveyed symbolic meanings to the participants, which prevented them from using a facemask in the post-SARS era. Using a facemask was widely perceived as a sign of being sick, and more than half of the participants perceived that using a facemask would subject them to stigmatization. One participant relayed her experience as follows:

I do not wear a facemask now, even though I am sick. Everyone will know that I am sick if I wear a facemask, and they will avoid me. It is noticeable when taking public transportation; no one is willing to sit next to anyone wearing a facemask. That’s true, and I experienced this before when using public transportation; people would walk away immediately if I wore a facemask. No one thinks that wearing a facemask when falling ill is a type of civic responsibility. Instead, people just get scared and avoid you when you are wearing a facemask.

[Female participant (P22), 29 years old]

Using a facemask was thus perceived as frightening to others, according to the participants’ experiences, and would be an invitation for social seclusion. Using a facemask often implied that one had become infected with a serious infection that might endanger the health of others. One participant indicated how she reacted to those who used a facemask as follows:

I think that those who wear a facemask must have gotten infected with a very serious infection such as tuberculosis. It is unnecessary for normal people to wear a facemask if they just catch a cold or flu, because most colds and flus are not serious at all, and will not kill people. Therefore, if a person needs to wear a facemask, I would assume that he or she must have gotten a very serious infection. I will avoid them, because they may make me very sick, or even kill me.

[Female participant (P30), 36 years old]

Perceiving that facemask use was chiefly for infection prevention, certain participants had used a facemask to prevent themselves from becoming infected. However, the experience of being teased prevented more than half of the participants from using a facemask in the post-SARS era. One participant shared the following common experience:

Some people are really bad; they will tease you if you wear a facemask. Just like my colleagues; they never wear a facemask when they are sick, and they never cover their nose and mouth when coughing or sneezing. Even worse, some really bad colleagues will cough and sneeze on you! I am afraid of being infected, so I wear a facemask because I cannot push them to wear one. However, these bad colleagues would tease me. Even in public transportation, I also wear a facemask if others around me keep coughing or sneezing. However, these selfish people would shoot me a dirty look, and some even cough and sneeze even harder! Because of these people, I always feel reluctant to wear a facemask, even though I am really afraid of being infected.

[Female participant (P19), 36 years old]

#### Facemask as a symbol associated with negative attributes for users

More than half of the participants stigmatized people who used facemasks in the post-SARS era. To these participants, those who use a facemask were widely misperceived as strange, and perhaps even mentally ill. One participant noted the following:

Other than those working in hospitals and clinics, I always feel that only strange and mentally ill people wear a facemask. Even though they may not really be mentally ill, I think they may have mysophobia. I have really seen some people with facemasks wearing gloves as well. They appear to be mysophobic, and their behaviors also appeared strange and to be displaying sickness. I tend to stay away from these people wearing facemasks, because I never know if they will go crazy suddenly.

[Male participant (P8), 34 years old]

The participants also popularly stigmatized those who used a facemask as criminals. The facemask was perceived as a tool used by criminals for concealing their faces. One participant expressed how he obtained such an impression from media reports as follows:

If you read the news reports and watch the news on TV, you’ll see that these criminals often love wearing facemasks. My immediate impression of these people wearing facemasks is that they are criminals. They wear a facemask because they “cannot see the light” [Cantonese slang metaphor indicating someone who has committed bad deeds], and they do not want to be recognized by others. If you behave well and if you can “see the light”, you do not need to wear a facemask to cover up your face.

[Female participant (P15), 25 years old]

The changing political environment of Hong Kong also influenced how the participants perceived the facemask usage behavior. The participants often associated those who used a facemask with rioters. One participant stated the following:

I dare not use a facemask now, because I do not want to be misunderstood as a member of Occupy Central [a political movement in Hong Kong that began in November 2014; also referred to as the Umbrella Movement]. Those who join Occupy Central and the violent protests wear facemasks at these activities, because they are afraid of being recognized, and “cannot see the light” to some extent. Facemasks are mainly used by violent protesters to cover their faces now. Therefore, I dare not use a facemask now, because I do not want to be misunderstood as a violent protester.

[Female participant (P25), 52 years old]

The participants also associated facemask use with people who had a low social status. Cleaners were often stereotyped as the most common social group who used facemasks, according to the participants’ perceptions. Therefore, the participants were reluctant to use a facemask because they did not want to be misidentified as cleaners, which was perceived as an occupation indicating a low social status. One participant stated the following:

It is uncommon for people to wear a facemask now, except for cleaners. Cleaners often wear a facemask on the streets. If I wear a facemask, I am afraid others will misunderstand that I am working as a cleaner, so I have not used a facemask for a long time, since [the] SARS [outbreak].

[Female participant (P12), 54 years old]

#### Facemask as a sign of weakness

Certain participants also perceived that wearing a facemask could convey an image to others that they were physically weak, making them reluctant to use one. This perception was particularly prevalent among male participants, because being weak violated the cultural expectations of men, who were assumed to be physically strong. One male participant indicated the following:

Wearing a facemask means that I am weak and afraid of bacteria and germs. But I am a man, and I think I am strong, so I do not need to wear a facemask. Even the government advises facemask usage only for those who are physically weak, such as elderly people, chronically ill patients, pregnant ladies, and children, who will need to wear a facemask during flu season. If I wear a facemask, I will lose face in front of my friends, and they will laugh at me for being so weak.

[Male participant (P36), 31 years old]

Using a facemask was also perceived as a sign of being scared of death for the participants. One participant indicated the following:

I think that only those who are afraid of death will wear a facemask. I am not afraid of death, and I think I am strong and healthy. Therefore, I do not think I need to use a facemask. I always think that it should not be too easy for one to die. If I wear a facemask, others may misunderstand that I am very weak and afraid of dying.

[Male participant (P32), 35 years old]

#### Facemask as a toxin to skin

The hesitation in using a facemask among the participants was also due to the popular perception of facemasks as a toxin for skin. Almost half of the participants expressed doubt regarding the quality, hygiene, and production process of facemasks. The use of artificial chemicals in facemask production also made the participants hesitant to use a facemask. One participant stated the following:

Wearing a facemask is bad for your skin, because you can never know whether the facemask production process is hygienic. Facemask production also involves a lot of chemicals, dyes, and so on, and they are harmful to your skin for sure. You may absorb these toxic materials into your body through your skin. Moreover, wearing a facemask can make me more susceptible to allergies and acne, so I have a lot of doubt regarding the quality of facemasks. When you use a facemask, you breathe in toxic materials. Anyway, I do not use facemasks, because I always think that they contain a lot of toxins.

[Female participant (P1), 32 years old]

The lack of trust on mainland China led certain participants to express a lack of confidence in the production locations of facemasks, preventing them from using a facemask. One participant stated the following:

I dare not wear a facemask, because you know that most facemasks are produced in mainland China. Even if you buy facemasks from Watson’s and Manning’s [drugstore chains], the quality cannot be ensured because these facemasks are made in China. Will you trust the quality of products that are made in China? I definitely won’t. The quality of products that are made in mainland China cannot be guaranteed, and they may contain a lot of toxins. These made-in-China facemasks may be made of “black-heart cotton” [contaminated materials]. Anyway, I do not have any confidence in products that are manufactured in China. I do not trust the quality of these facemasks. If facemasks were produced in other overseas countries such as Japan or Western countries, I would feel safe using them. However, it is very difficult to buy these facemasks.

[Female participant (P7), 44 years old]

## Discussion

The SARS outbreak in 2003 marked a critical turning point in the development of infection control and public health in Hong Kong. One of the most remarkable outcomes in terms of infection control after the SARS outbreak was the awareness of facemask usage in Hong Kong, not only at the clinical level but also at the community level. Although Hong Kong citizens have become familiar with facemask use since the SARS outbreak, the sociocultural meanings of facemasks have been undergoing constant change, which resulted in the participants experiencing hesitation to use facemasks in the post-SARS era. The old meanings associated with facemasks that had developed during the SARS outbreak failed to be sustained in the post-SARS era. Changes in the sociocultural meanings of the facemask not only influenced the participants’ perceptions of facemask but also influenced their views of those who used facemasks, which ultimately affected their health behavior, reducing the likelihood of their facemask use in the post-SARS era. Moreover, the negative perceptions associated with facemasks also contributed to the changes in the sociocultural implications of facemasks in post-SARS Hong Kong. As indicated by the participants, the shifting meanings of facemasks could be explained by the violation of society’s norms and ideologies, by the violation of the traditional Chinese cultural beliefs on healing, and by the projection of the difficult relationship between mainland China and Hong Kong.

The experiences of the participants since the SARS outbreak are critical in the shaping of their perceptions of facemasks. All of the participants had firsthand experience with SARS in Hong Kong during the SARS outbreak, and they had differing experiences during the outbreak. Certain participants lost their family members during the outbreak because of SARS, some other participants had family members who were infected with SARS, and others were not directly affected by SARS but were frustrated by it. Although they had different experiences of the SARS outbreak, they had all used a facemask throughout the outbreak because of fear and sociocultural forces. When the post-SARS era approached, however, the different experiences of the participants influenced their perceptions of facemask use. The lack of a direct experience with an epidemic in the post-SARS era for many participants, in addition to the social changes, made them disregard the infection prevention value of facemasks, thereby contributing to the shifting sociocultural meanings of facemasks in the post-SARS era.

The extent of the participants’ experiences with the SARS outbreak also significantly influenced how they perceived facemask usage behavior in the post-SARS era. Those who encountered more direct and traumatic experience in the SARS outbreak and the older had a more positive attitude toward facemask use compared with younger participants, who perceived facemask use to be more negative in the post-SARS era. Because the younger participants were unaware of what had transpired during the SARS outbreak because of their young age (certain participants were in primary school during the outbreak), they did not have a clear understanding of why they were required to use a facemask during the outbreak. Many of them had used a facemask during the SARS outbreak, merely because of the control of social institutional forces such as their parents, teachers, and school. Consequently, without a strong experience, these younger participants did not have the cultural foundations to understand facemask usage behavior in the post-SARS era. By contrast, older participants typically had a more positive attitude toward facemask use in the post-SARS era, even though they did not use a facemask in the post-SARS era. The older participants usually displayed more intense feelings and relayed such experiences pertaining to the SARS outbreak, leading to a stronger understanding regarding the importance of using a facemask in the post-SARS era.

The only meaning of facemasks that remained static during and after the SARS outbreak in Hong Kong was their purpose of practical infection prevention. Although according to clinical guidelines facemasks should theoretically be used predominantly by patients with respiratory tract infections to prevent them from infecting others [16–18], the participants commonly perceived the opposite: Facemasks were perceived as a tool for preventing themselves from becoming infected during the SARS outbreak and in the post-SARS era. Such an embedded perceived purpose of facemasks thus explains the participants’ reluctance to use a facemask, despite their experiences with respiratory tract symptoms in the post-SARS era.

Symbolic meanings are often constructed for practical use. In addition to the practical purpose of infection prevention, facemasks also conveyed critical symbolic meanings to participants during the SARS outbreak. These symbolic meanings were largely constructed by a social authority—health care providers—resulting in the construction of a new social norm. The health care profession is a form of social institution [19]; the public’s trust in health care providers [19] often enables them to assume an authoritative role in most societies, making them a key social group with authority and social power over the creation of new social norms and the implementation of social control [20]. During the SARS outbreak, health care providers occupied an even more prestigious position, and they were portrayed as social heroes in the battle against SARS [21]. Thus, they held even more power in the construction of this new social norm. They used facemasks in media appearances [22], and they encouraged the public to use facemasks in the community during the SARS outbreak [23]. Therefore, other than stethoscopes and white coats, facemasks were an additional entity associated with health care providers during the SARS outbreak, causing facemasks to become another critical symbol representing health care providers at the time.

The symbolic association of facemasks with health care providers influenced the participants’ use of facemasks during the SARS outbreak at two levels. First, the use of facemasks among health care providers on different social occasions and in media appearances was crucial for the construction of this new social norm and for normalizing the use of facemasks, because of their social power. Health care professionals, as a social institution, allowed them to exercise social control with respect to facemask use in the community, which motivated the participants to use a facemask during the SARS outbreak. Second, the participants used facemasks as a form of symbolic support for health care providers. Because facemasks were perceived as a tool for infection prevention, using a facemask did not simply prevent the participants from becoming infected, but by so doing, it also implied a show of support for health care providers in an attempt to reduce their workload and the burden on the health care system. Using a facemask was akin to a symbolic declaration that they were committed to reducing the burden on the health care system by preventing themselves from becoming infected. Such symbolic support for health care providers through facemask use extended further to the social implication of displaying civic responsibility. These sociocultural processes hence contributed to the symbolic construction of the facemask during the SARS outbreak.

In addition to the health care providers, the mass media played a critical role in the construction of such symbolic implications by reinforcing the new social norm of using facemasks in public areas during the SARS outbreak. Chinese-language media devoted significantly more space to reporting news on SARS daily [24]. Photographs showing people using facemasks occupied the newspaper headlines every day [24]. People who failed to use a facemask in public areas were represented as abnormal in news reports. Those who were infected with SARS were condemned as spreading the virus in these reports. “Super virus spreaders” [25], for example, was the popular term used by the mass media for representing patients infected with SARS. Such a sensational reporting style with moral judgment and condemnation thus made the infection of SARS antisocial. Consequently, people were afraid of becoming infected with SARS, and this constructed, normalized, and reinforced the new social norm of using facemasks in public spaces. Those who failed to use a facemask were perceived as antisocial, and thus, were discriminated against. The construction of such a social norm portrayed facemasks and reinforced them as a sign of civic responsibility on another level: The purpose of using a facemask was represented as not only for the user’s benefit but also for the good of the community and as a show of support.

The perception of the predominant use of facemasks for infection prevention also made facemasks a critical tool for the participants to achieve a sense of control and security during the SARS outbreak. The high mortality rate and unknown transmission route of SARS made it a mysterious disease to most Hong Kong citizens during the outbreak [24]. In the social atmosphere that was filled with uncertainty, using a facemask was the only measure for the participants to protect themselves. Consequently, the use of a facemask for infection prevention was further reinforced, which deterred the participants from learning the other critical use of facemasks, that of preventing the transmission of infectious diseases to others. Hence, the participants had been unaware of using a facemask when they experienced respiratory symptoms, both during and after the SARS outbreak.

The perception of the use of facemasks for infection prevention was thus embedded in the participants’ minds during the SARS outbreak and continued into the post-SARS era. The embedded belief in the purpose of the facemask for infection prevention served as an underlying factor for cultivating a shift in the meanings associated with facemasks in post-SARS Hong Kong. Although using a facemask in public areas was constructed as a new social norm in Hong Kong during the SARS outbreak, and such behavior was constructed to be a symbol of support for health care providers and a display of civic responsibilities at the time, these social norms and meanings have been gradually diminishing in post-SARS Hong Kong. The absence of an epidemic outbreak that is similar to that of SARS as well as the physical discomfort when using a facemask were the immediate factors, but other sociocultural values as well as the changes of social and political environment also interlocked to contribute to the shift in the meanings of facemasks in the post-SARS era.

The sociocultural meanings of facemasks have shifted from positive to negative in post-SARS Hong Kong. Without the presence of a significant epidemic outbreak, the embedded stereotypes toward facemask among the participants thus reoccupied their perceptions. The embedded traditional Chinese cultural belief regarding infectious diseases and healing acted as a considerable obstacle in preventing almost all the participants from using a facemask in post-SARS Hong Kong, resulting in a failure to sustain facemask usage for preventing the spread of infectious diseases. Traditional Chinese medicine (TCM) is the most popular ethnic medical system in Hong Kong, existing alongside the mainstream biomedicine. Influenced by views in TCM, the popular belief of “virus and bacteria must be released from the body for recovery” was deeply ingrained in the participants’ minds. The use of a facemask, however, violates this TCM ideology, because a facemask prevents viruses and bacteria from escaping the body, which is perceived as hindering recovery from the participants’ perceptions. Consequently, the use of a facemask was not welcome by the participants, due to this violation of traditional Chinese cultural belief on healing. This contributed as an underlying factor to the shift in meaning of facemasks, resulting in the failure to sustain the values associated with facemasks that had developed during the SARS outbreak.

To reinforce such traditional cultural values on disease and healing belief, the participants thus recreated the new meanings of facemask that were consistent with these long existing cultural values in the post-SARS era. Using a facemask in the post-SARS era was no longer perceived as a sign of civic responsibility; instead, such health behaviors were stigmatized and constructed to be correlated with a person’s negative attributes. Using a facemask was also correlated with a person’s low social standing and was even correlated with antisocial people such as criminals and violent protesters. With these negative attributes attached to facemasks, the sociocultural meanings of facemasks have shifted from positive to negative, which further deterred the participants from using a facemask in the post-SARS era.

Although the participants still perceived infection prevention to be the main purpose of using a facemask in post-SARS Hong Kong, and although the participants commonly believed that facemasks were for use by healthy people to prevent themselves from becoming infected, the intention to prevent infection has been associated with negative attributes after the SARS outbreak. Those who used a facemask in public areas in the post-SARS era were stigmatized by the participants with different biases (e.g., strangeness, mental illness, and mysophobia). These stigmas violated the sociocultural ideals of Hong Kong. Patients with psychiatric diseases have been stigmatized in Hong Kong as, for instance, crazy, dangerous, and violent [26], and were thus perceived to pose a hidden risk to society [27]. Those with mysophobia were often stigmatized as mentally ill as well [28]. To avoid being stigmatized as mentally ill, the participants were deterred from using a facemask.

In addition, other negative attributes associated with facemasks in the post-SARS era also violated the dominant ideologies of Hong Kong. Because of the mass media, according to the participants’ perceptions, the use of a facemask was often associated with people who were antisocial (e.g., criminals and violent protesters). Indeed, antisocial deviants in Hong Kong often used facemasks intentionally in media coverage. Hence, the use of a facemask was widely perceived as a tool for avoidance of being recognized for engaging in actions that violated the law. Under the further reinforcement of the mass media, the participants thus constructed a negative association with the use of facemasks. The negative perception of those who used a facemask was reinforced further after the Umbrella Movement in late 2014, in which the protesters went on riot with facemasks. In a society with a heavy emphasis on law and order such as that of Hong Kong, the participants were deterred from pursuing this health behavior because they did not wish to be misperceived either as antisocial criminals or violent protesters. The mass media and the changing social and political environment in Hong Kong, thus, have contributed to the shifting social and cultural meanings of facemask in post-SARS era.

Moreover, facemask use was often associated with people who were low on the social hierarchy. To the participants, cleaners were another social group who often used facemasks. Because cleaners were perceived to hold a low social status in Hong Kong, this violated the capitalist ideology of Hong Kong, which strongly emphasizes wealth and a high social status. All of these negative attributes were constructed to be correlated with the use of a facemask in the post-SARS era, which prevented the participants from using a facemask. The sociocultural meanings of the facemask have thus been undergoing continuous change in Hong Kong. These changes influenced the participants’ perceptions of the facemask and hindered their adoption of facemask usage behavior.

Although facemasks are still perceived as a tool for infection prevention after the SARS outbreak, the participants also held contradictory views on the use of facemasks pertaining to their use as an obligation for patients with infectious diseases. However, patients using a facemask were often subjected to a stronger stigma according to the participants, because these patients were often viewed as suffering from serious infectious diseases that could endanger others’ health. Consequently, using a facemask in public was frequently an invitation for social seclusion. To avoid being isolated, the participants were thus deterred from using a facemask in post-SARS Hong Kong.

The fragile relationship and sociopolitical tensions between Hong Kong and mainland China, in addition to the widespread distrust among Hong Kong citizens of mainland China, also led to participant concerns regarding the safety of and confidence in facemasks, preventing them from using facemasks in post-SARS Hong Kong. This subtle display was made tangible and reinforced by the mass media through news reports. Because of the numerous media reports regarding the unscrupulous and unhygienic production process of products made in China, the participants held a high degree of distrust in mainland Chinese products. Because most facemasks were assumed to be produced in mainland China, the participants experienced doubt and lacked confidence in the quality and hygiene of the facemasks. At the same time, mass media had been kept reinforcing about “black-heart cotton” in their reports about mainland Chinese products, making the participants commonly perceived that the facemask could itself contain toxins, and labelled these facemasks as making of “black-heart cotton”. As shown by the participants, such sociopolitical tension as well as the cultural differences between Hong Kong and mainland China thus projected on and manifested in the participants’ doubt regarding the safety of facemasks, which aroused their sense of insecurity. The failure to sustain the sociocultural implications of facemasks in post-SARS Hong Kong can thus also be explained by the sociopolitical tension between Hong Kong and mainland China.

Traditional Chinese gender values could explain the participants' gender differences of their acceptance of using a facemask in the post-SARS era. Male participants were particularly more reluctant to use a facemask, because facemask usage behavior violated the sociocultural expectations of men in Chinese societies. To these participants, using a facemask was widely perceived as a sign of weakness, thereby violating the sociocultural expectations of men, who should be “strong” and “brave.” The violation of traditional Chinese cultural expectations regarding men thus served as a considerable obstacle for the male participants in using a facemask in post-SARS Hong Kong.

### Implications to public health

Since the SARS outbreak, there had been several infectious disease outbreaks in Hong Kong, such as the Influenza A (H1N1) pandemic in 2009, and many more Influenza outbreaks in the winter peak seasons every year. Other pandemics such as avian influenza and MERS have also been threatening the public health of Hong Kong. However, the importance of the preventive health behavior adoption of facemask using has been diminishing. The shifting sociocultural meanings of facemask in Hong Kong since the SARS outbreak, as demonstrated by the participants, can explain this preventive health behavioral change on the community level. The shifting sociocultural meanings of using facemask can demotivate people in adopting the facemask using behavior, making infection control as more difficult to achieve during epidemic outbreaks, conveying significant implications for the public health and infection control in the post-SARS era. Facemask using has been documented as having significant clinical importance in infection prevention [2, 3]; encouraging people using a facemask is thus one of the remarkable public health measures on epidemic containment. Therefore, one of the key directions of public health and infection prevention policy in future is suggested to adopt a culturally sensitive education approach, educating the general public about the positive aspects of facemask using behavior.

### Limitations

This study had limitations. All of the participants were sampled from one private-practice primary care clinic. Because the fee for attending a private clinic is often higher than for public clinics, the socioeconomic status of the participants in this study was assumed to be higher. Therefore, these findings mainly reflected the perceptions of those with a higher socioeconomic background. People with a lower socioeconomic status may be excluded from generalization. Future research with more varied field sites will provide a more holistic understanding.

## Conclusion

Facemask use should thus not be simply viewed as a public health behavior; instead, sociocultural reasons are intertwined to explain people’s usage or avoidance of facemasks. Because of the negative stereotypes associated with the use of facemasks in post-SARS Hong Kong, the sociocultural meanings of facemasks have undergone continuous change, from positive to negative. Because of the changes in social needs, the meanings of facemasks that had developed during the SARS outbreak could not be sustained in the post-SARS era. During the SARS outbreak, the social functions of the facemask were significant not only in terms of providing a practical approach to infection prevention, but also in providing symbolic uses for obtaining a sense of control and security, fulfilling the new social norm, and displaying civic responsibility and support for health care providers. To fulfill these social needs, the sociocultural meanings of facemasks were thus constructed to be positive. However, such social needs were lacking in the post-SARS era, leading to changes in the social functions and sociocultural meanings of facemasks. The changes to the social and political environment generated a negative image of facemask usage, preventing the participants from pursuing this health behavior. The experiences of the participants thus enabled a holistic understanding regarding the perceptions of facemask use. The shifting sociocultural meanings of facemask use in Hong Kong, however, can impose a potential risk to the public health of Hong Kong citizens in case of a future epidemic outbreak. Witnessing younger participants tending to hold more negative perceptions of facemask usage in the post-SARS era was alarming. These findings have critical implications for health authorities in designing a culturally responsive infection prevention and facemask usage policy in the future.

## References

[1] World Health Organization. Summary of probable SARS cases with onset of illness from 1 November 2002 to 31 July 2003. 2003. http://www.who.int/csr/sars/country/table2003_09_23/en/. Accessed January 5, 2015.

[2] Sim SW, Moey KS, Tan NC (2014). The use of facemasks to prevent respiratory infection: a literature review in the context of the health belief model. Singapore Med J.

[3] Suess T, Remschmidt C, Schink SB, Schweiger B, Nitsche A, Schroeder K, Doellinger J, Milde J, Haas W, Koehler I, Krause G, Buchholz U. The role of facemasks and hand hygiene in the prevention of influenza transmission in households: results from a cluster randomised trial; Berlin, Germany, 2009–2011. BMC Infect Dis. 2012;12:26. doi:10.1186/1471-2334-12-26. 10.1186/1471-2334-12-26 PMC3285078 22280120

[4] American University of Beirut. https://www.aub.edu.lb/fm/shbpp/white/Documents/drsamiratwe.pdf. Accessed January 8, 2015.

[5] Rice T (2010). ‘The hallmark of a doctor’: the stethoscope and the making of medical identity. J Mater Cult.

[6] Richardson ME (2015). An evidence-based practice case study: white coat hypertension. Plast Surg Nurs.

[7] Apple Daily. 韓教授罵港生:你國家是沙士源頭 [Korean professor scolded Hong Kong students, “Your country was the originating place of SARS”]. 2015. http://hk.apple.nextmedia.com/news/art/20150612/19181547. Accessed July 5, 2015.

[8] Liberty Times Net.「不要帶敏感情緒來南韓」港生戴口罩遭教授斥責 [Hong Kong students with facemasks were scolded by Korean professors, “Don’t bring along the sensitive emotion to South Korea”]. 2015. http://news.ltn.com.tw/news/world/breakingnews/1343654. Accessed July 5, 2015.

[9] Oriental Daily. 新沙士:港人遊韓戴口罩遭學生大聲咳嘲笑 [New SARS: Hong Kong tourists with facemasks were teased and being paid with cough by Korean students]. 2015. http://hk.on.cc/hk/bkn/cnt/news/20150608/bkn-20150608203051727-0608_00822_001.html. Accessed July 5, 2015.

[10] Television Broadcasting Company Limited. 本月餘下南韓團或取消 交流生批韓缺防疫意識 [Tours to Korea will be cancelled. Exchange students from Hong Kong criticized South Koreans as lacking awareness in infection control.]. 2015. http://news.tvb.com/local/5575af7d6db28c594d000003/. Accessed July 5, 2015.

[11] Francis JJ, Johnston M, Robertson C, Glidewell L, Entwistle V, Eccles MP, Grimshaw JM. What is an adequate sample size? operationalising data saturation for theory-based interview studies. Psychol Health. 2010;25(10):1229–45. 10.1080/08870440903194015 20204937

[12] Given, L.M. The Sage Encyclopedia of Qualitative Research Methods. 2008. doi: http://dx.doi.org/10.4135/9781412963909.

[13] Green J, Thorogood N (2004). Qualitative methods for health research.

[14] Parse RR (2001). Qualitative inquiry: the path of sciencing.

[15] Bernard HR (2002). Research methods in anthropology: qualitative and quantitative approaches.

[16] Centre for Health Protection, Department of Health, The Government of the Hong Kong Special Administrative Region. CHP’s review and outlook of infectious diseases. 2006. http://www.chp.gov.hk/en/view_content/5365.html. Accessed February 2, 2015.

[17] World Health Organization. Procedures for prevention and management of probable cases of SARS on international cargo vessels. 2003. http://www.who.int/csr/sars/travel/maritime/en/. Accessed February 3, 2015.

[18] World Health Organization. Tuberculosis and air travel: Guidelines for prevention and control. Third edition. 2008. http://www.who.int/tb/publications/2008/WHO_HTM_TB_2008.399_eng.pdf?ua=1. Accessed February 4, 2015. 23785743

[19] Gilson L (2003). Trust and the development of health care as a social institution. Soc Sci Med.

[20] Loustaunau MO, Sobo EJ (1997). The cultural context of health, illness, and medicine.

[21] Global Times. HK’s “SARS heros” share reflections a decade after deadly virus outbreak. 2013. http://www.globaltimes.cn/content/762844.shtml. Accessed February 6, 2015.

[22] The Chinese University of Hong Kong. Fighting SARS: We care we serve. Clinical and scientific work. 2003. http://www.cuhk.edu.hk/health_promote_protect/sars_care/menu2.htm. Accessed January 19, 2015.

[23] Sina News Hong Kong. 鎖匙按電梯掣 8A主診醫生提出 [A doctor of ward 8A in Prince of Wales Hospital suggests using keys to press lift button]. 2013. http://news.sina.com.hk/news/20130221/-1797-2900053/1.html.Accessed February 13, 2015.

[24] Loh C, Civic Exchange (2004). At the epicentre: Hong Kong and the SARS outbreak.

[25] eElderly. 社評:應對疫症硬道理 寧可做多勿做少 [Editorial: Be forceful against epidemic, doing more is always better than doing less]. 2015. http://www.e123.hk/ElderlyPro/details/405505/2/. Accessed August 5, 2015.

[26] The Mental Health Association of Hong Kong. 精神病的誤解與事實 [The misunderstanding and the truth about psychiatric disease]. 2015. http://www.mhahk.org.hk/chi/sub4_1_info_b1_2.htm. Accessed August 5, 2015.

[27] School of Journalism and Communication, The Chinese University of Hong Kong. 別再歧視我!精神病康復者的心聲. [Do not discriminate against me! The voice of recovering patients with psychiatric disease]. 1997. In U-Beat Magazine. http://www.com.cuhk.edu.hk/ubeat_past/970515/15mental.htm. Accessed April 6, 2015.

[28] Apple Daily. 洗手細菌洗唔走越潔癖越致命 [Washing hands cannot clear all bacteria. Mysophobia can kill]. 2010. http://hk.apple.nextmedia.com/supplement/health/art/20100611/14122137. Accessed February 15, 2015.

